# Generation of Budoids: 3D Multilineage Limb Models From Mouse Embryonic Stem Cells

**DOI:** 10.21769/BioProtoc.5808

**Published:** 2026-09-05

**Authors:** Kelly Hu, Can Aztekin

**Affiliations:** Friedrich Miescher Laboratory of the Max Planck Society, Tübingen, Germany

**Keywords:** Signaling centers, Mouse embryonic stem cells, Small-molecule differentiation, Organoids, Limb development

## Abstract

Limb development requires the coordination of multiple cell types, including the limb bud mesoderm and surface ectoderm, by the apical ectodermal ridge (AER), a specialized signaling center secreting numerous morphogens. Characterizing these cell–cell interactions is crucial for understanding limb morphogenesis, but they are challenging to study in vivo. Furthermore, existing in vitro models do not capture the multilineage complexity of the limb. We recently developed a robust 7-day differentiation protocol using mouse embryonic stem cells (mESCs) to generate heterogeneous cultures containing cells with characteristics of the limb bud mesoderm, surface ectoderm, and AER. Dissociating and reaggregating these cultures in low attachment 96-well plates forms budoids, organoids that display certain limb bud–like features. Budoids undergo chondrogenesis-mediated symmetry breaking and elongation within 5 days of culture. Altogether, our protocols have enabled the study of cell–cell interactions in limb development and provide an easily scalable model adaptable for various applications, including drug testing and congenital disorder modeling.

Key features

• Induction of heterogeneous 2D cultures containing three main cell types of the developing limb bud from mESCs.

• Free-floating budoid cultures achieve 3D morphogenesis without manual manipulation or embedding.

• Highly scalable protocol, per differentiation experiment yielding > 9 × 10^6^ cells, sufficient for generating >1,000 budoids.

## Graphical overview


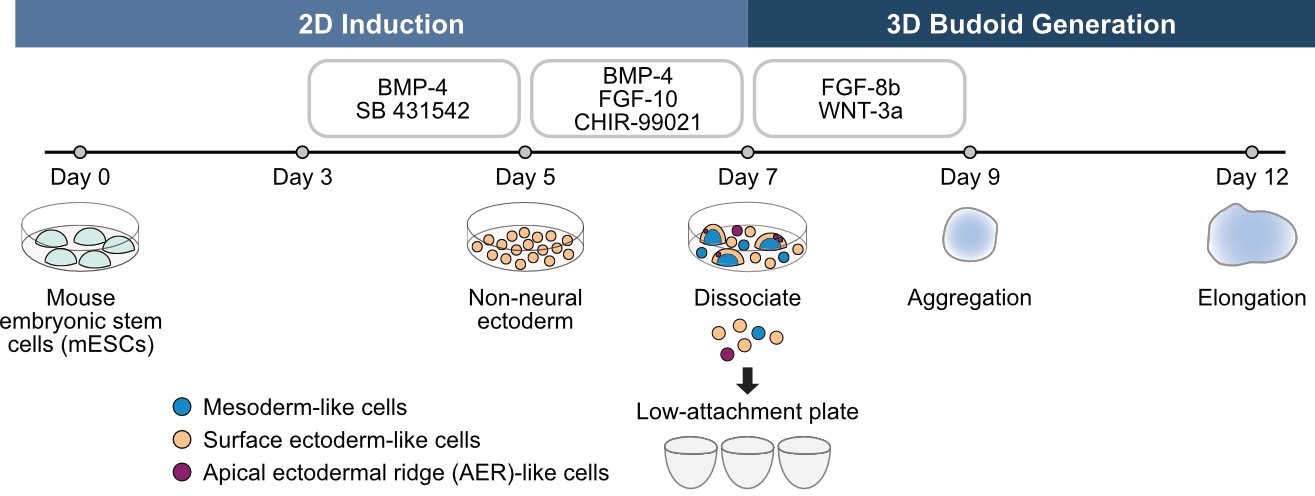


## Background

The developing limb bud has long been used as a model for studying patterning and morphogenesis. Notably, limb morphogenesis relies on multilineage interactions between limb bud cell types, including the limb bud mesoderm, surface ectoderm, and the apical ectodermal ridge (AER) [1–3]. The AER, which forms at the distal tip of the limb bud as a thickened ectoderm [4,5], is a specialized signaling center that dynamically coordinates the proliferation and differentiation of the other limb bud cell types via various secreted morphogens [4,6–8]. Historically, the AER has been examined in model organisms via tissue-level excision and grafting experiments [3,9–11] as well as gene inactivation approaches [12–14], but its interactions remain largely uncharacterized at the cellular level. Previous in vitro limb development models [15–17] mainly focused on generating mesoderm. Therefore, new models are needed to decipher cell–cell interactions involved in limb morphogenesis.

Organoids are simplified stem cell–based models that recapitulate certain features of a given organ and development [18]. Key to their utility is the ease with which aspects of in vivo–like processes can be observed and subject to chemical, genetic, and mechanical perturbations. A mouse limb bud organoid system was previously proposed [19], yet it showed limited changes in morphology, required laborious manual processing, and lacked complete cellular characterization. To enable the study of individual limb cell populations, we established a mouse embryonic stem cell (mESC)-based differentiation protocol applicable for the mass production of cell types with limb bud mesoderm–like, surface ectoderm–like, and AER-like characteristics. By dissociating and reaggregating these cultures in U-bottom microplates, we generate organoids that provide insights into how limb bud cell types self-organize and interact to achieve morphogenesis. Moreover, our budoid protocol can be extended to limb bud cells from mouse embryos, instead of mESCs, producing in vivo–derived limb models. Thus, these organoids, termed budoids, facilitate 3D limb development modeling at an unprecedented scale. Diverse possibilities are open for future studies with our 2D induction and budoid protocols, including drug testing and congenital disorder modeling. Here, we present a detailed protocol for these methodologies.

## Materials and reagents

**Biological materials**

1. EmbryoMax^®^ embryonic stem cell line, strain 129/SVEV, passage 11 (Merck, catalog number: CMTI-1; RRID: CVCL_GS41)

**Reagents**

1. Dulbecco’s modified Eagle’s medium (DMEM), high glucose, GlutaMAX^TM^ supplement (Gibco, catalog number: 61965026)

2. Embryonic stem cell fetal bovine serum (FBS), qualified, US origin (Gibco, catalog number: 16141079)

3. Penicillin-Streptomycin (10,000 U/mL) (Gibco, catalog number: 15140122)

4. Minimal essential medium (MEM) non-essential amino acids solution (100×) (Gibco, catalog number: 11140035)

5. Sodium pyruvate (100 mM) (Gibco, catalog number: 11360070)

6. 2-Mercaptoethanol (50 mM) (Gibco, catalog number: 31350010)

7. EmbryoMax^®^ 0.1% gelatin solution (Merck, catalog number: ES-006-B)

8. Phosphate-buffered saline (PBS), pH 7.4 (Gibco, catalog number: 10010015)

9. Trypsin-EDTA (0.25%), phenol red (Gibco, catalog number: 25200072)

10. Bovine albumin fraction V (7.5% solution) (Gibco, catalog number: 15260037)

11. UltraPure^TM^ DNase/RNase-free distilled water (Thermo Fisher Scientific, catalog number: 10977035)

12. LIF (in-house preparation) or human LIF, animal-free recombinant protein, PeproTech^®^ (Gibco, catalog number: AF-300-05-25UG) (used interchangeably)

13. PD0325901 (Mirdametinib) (Selleckchem, catalog number: S1036), product format: 10 mM (1 mL in DMSO)

14. CHIR-99021 (CT99021) (ApexBio, catalog number: A3011), product format: 10 mM (in 1 mL DMSO) or GSK-3 Inhibitor XVI (Calbiochem, catalog number: 361559) (used interchangeably)

15. Glasgow’s MEM (GMEM) (Gibco, catalog number: 11710035)

16. KnockOut^TM^ serum replacement (Gibco, catalog number: 10828010)

17. Normocin^®^ antimicrobial reagent (InvivoGen, catalog number: ant-nr-05)

18. Human BMP-4 recombinant protein, PeproTech^®^ (Gibco, catalog number: 120-05ET)

19. SB 431542 (ApexBio, catalog number: A8249), product format: 10 mM (1 mL in DMSO)

20. Human FGF-10, animal-free recombinant protein, PeproTech^®^ (Gibco, catalog number: AF-100-26)

21. Human/mouse FGF-8b recombinant protein, PeproTech^®^ (Gibco, catalog number: 100-25)

22. Mouse Wnt-3a recombinant protein, PeproTech^®^ (Gibco, catalog number: 315-20)

23. Ethanol 70% v/v

**Solutions**

1. DMEM base medium (see Recipes)

2. DMEM+++ medium (see Recipes)

3. Non-neural ectoderm (NNE) medium (see Recipes)

4. Induction medium I (d3) (see Recipes)

5. Induction medium II (d5) (see Recipes)

6. Budoid generation media (d7) (see Recipes)

**Recipes**

See General notes 1 and 2 prior to beginning.

**1. DMEM base medium**

**Table:** 

Reagent	Final concentration	Volume (for 500 mL)
DMEM, high glucose, GlutaMAX^TM^ supplement	86.8% v/v	434 mL
Embryonic stem cell FBS	10% v/v	50 mL
Penicillin-streptomycin (10,000 U/mL)	1% v/v	5 mL
MEM non-essential amino acids solution (100×)	1×	5 mL
Sodium pyruvate (100 mM)	1 mM	5 mL
2-Mercaptoethanol (50 mM)	0.1 mM	1 mL

*Note: Prior to use, thaw embryonic stem cell FBS overnight at 4 °C, heat-inactivate for 30 min in a 56 °C water bath, and store in single-use aliquots at -20 °C. To prepare DMEM base medium, remove 66 mL of media from a 500 mL bottle of DMEM. Add all components and filter-sterilize the medium (e.g., with a Stericup vacuum filter). Store media at 4 °C for up to 1 month. Bring to 37 °C before use.*

**2. DMEM+++ medium**

**Table:** 

Reagent	Final concentration	Volume (for 50 mL)
DMEM base medium	100% v/v	50 mL
Human LIF, animal-free recombinant protein (100 μg/mL)	100 ng/mL	50 μL
PD0325901 (2 mM)	1 μM	25 μL
CHIR-99021 (CT99021) (3 mM)	3 μM	50 μL

*Note: Prepare the medium in a 50 mL conical tube. Store at 4 °C for up to 1 week. Bring to 37 °C before use.*

**3. NNE medium**

**Table:** 

Reagent	Final concentration	Volume (for 50 mL)
GMEM	96% v/v	48 mL
KnockOut^TM^ serum replacement	1.5% v/v	750 μL
MEM non-essential amino acids solution (100×)	1% v/v	500 μL
Sodium pyruvate (100 mM)	1 mM	500 μL
Normocin^®^ antimicrobial reagent	0.2% v/v	100 μL
2-Mercaptoethanol (50 mM)	91 μM	91 μL

*Note: For optimal stability, protect all components from light. Prepare single-use aliquots of KnockOut^TM^ serum replacement and Normocin and store at -20 °C to avoid freeze-thaw cycles. Store media at 4 °C for up to 1 week, protected from light. Bring to room temperature before use.*

**4. Induction medium I (d3)**

**Table:** 

Reagent	Final concentration	Volume (per 12-well plate)
NNE medium	100% v/v	12.5 mL
Human BMP-4 recombinant protein (100 μg/mL)	100 ng/mL	12.5 μL
SB 431542 (2 mM)	1 μM	6.25 μL

*Note: Prepare fresh at the time of use. Make an aliquot of the desired volume of NNE in a conical tube and bring to room temperature before use, protected from light.*

**5. Induction medium II (d5)**

**Table:** 

Reagent	Final concentration	Volume (per 12-well plate)
NNE medium	100% v/v	12.5 mL
Human BMP-4 recombinant protein (100 μg/mL)	100 ng/mL	12.5 μL
Human FGF-10, animal-free recombinant protein (100 μg/mL)	100 ng/mL	12.5 μL
CHIR-99021 (CT99021) (3 mM)	3 μM	12.5 μL

*Note: Prepare fresh at the time of use. Make an aliquot of the desired volume of NNE in a conical tube and bring to room temperature before use, protected from light.*

**6. Budoid generation media (d7)**

**Table:** 

Reagent	Final concentration	Volume (per 60 budoids)
NNE medium	100% v/v	7 mL
Human/mouse FGF-8b recombinant protein (100 μg/mL)	450 ng/mL	31.5 μL
Mouse Wnt-3a recombinant protein (100 μg/mL)	300 ng/mL	21 μL

*Note: Prepare fresh at the time of use. Make an aliquot of the desired volume of NNE in a conical tube and bring to room temperature before use, protected from light.*

**Laboratory supplies**

1. Nunc^TM^ cell-culture-treated multidishes (6-well plate) (Thermo Fisher Scientific, catalog number: 140675)

2. Nunc^TM^ cell-culture-treated multidishes (12-well plate) (Thermo Fisher Scientific, catalog number: 150628)

3. Nunclon^TM^ Sphera^TM^ 96-well, Nunclon Sphera-treated, U-shaped-bottom microplate (Thermo Fisher Scientific, catalog number: 174929)

4. 1,000 μL XL graduated TipOne^®^ filter tip (StarLab, catalog number: S1122-1830)

5. 200 μL graduated TipOne^®^ filter tip (StarLab, catalog number: S1120-8810)

6. 20 μL beveled TipOne^®^ filter tip (StarLab, catalog number: S1120-1810)

7. 10/20 μL XL graduated TipOne^®^ filter tip (StarLab, catalog number: S1120-3810)

8. 200 μL TipOne^®^ tip, yellow non-filter (StarLab, catalog number: S1111-0706)

9. 5 mL Stripette^TM^ serological pipettes (Corning, catalog number: 4487)

10. 10 mL Stripette^TM^ serological pipettes (Corning, catalog number: 4488)

11. 25 mL Stripette^TM^ serological pipettes (Corning, catalog number: 4489)

12. 50 mL Stripette^TM^ serological pipettes (Corning, catalog number: 4490)

13. Countess^TM^ cell counting chamber slides with trypan blue solution (Thermo Fisher Scientific, catalog number: C10228)

14. Falcon^®^ 50 mL high-clarity PP centrifuge tube (Corning, catalog number: 352070)

15. Falcon^®^ 15 mL high-clarity PP centrifuge tube (Corning, catalog number: 352096)

16. Reaction tube, 2 mL, PP (Sarstedt, catalog number: 72.691)

17. 50 mL reagent reservoir, sterilized (Biotix, catalog number: SR-0050-5SC)

18. Pasteur pipettes without cotton plug, 2 mL, 150 mm (Carl Roth, catalog number: 4518) (for aspirator system)

19. Millipore^®^ Stericup^®^ quick-release vacuum filtration system (Merck, catalog number: S2GPU05RE)

20. Vented Millex^TM^-GV filter unit (sterile), pore size 0.22 μm (Merck, catalog number: SLGVV255F)

21. B. Braun Injekt^®^ Luer-Lock, 2-part disposable syringe (Braun, catalog number: 4606710V) (for reconstitution)

## Equipment

1. Herasafe^TM^ KS, Class II Biological Safety Cabinet (Thermo Fisher Scientific, catalog number: 51022515)

2. Heracell^TM^ VIOS CO_2_ Incubator, 165 L (Thermo Fisher Scientific, catalog number: 50145502)

3. Eppendorf^®^ Centrifuge 5910 Ri G with Rotor S-4xUniversal (Thermo Fisher Scientific, catalog number: EP5943000106)

4. Olympus^®^ CKX53 cell culture microscope with EP50 digital camera (Evident, catalog number: ckx53)

5. Grant Instruments^TM^ SUB Aqua Pro Water Bath with heat transfer beads (Grant Instruments, catalog number: 15187025)

6. Countess^®^ II FL Automated Cell Counter (Thermo Fisher Scientific, catalog number: AMQAF1000)

7. VACUSAFE Aspiration System (INTEGRA Biosciences, catalog number: 158320)

8. Eppendorf Research^®^ plus, 4-pack (Eppendorf, catalog number: 3123000950)

9. Eppendorf Research^®^ plus Move It^®^ (Eppendorf, catalog number: 3125000176)

10. Eppendorf^®^ Easypet^®^ 3 Electronic Pipette Controller (Eppendorf, catalog number: 4430000018)

11. Refrigerator (4 °C)

12. Freezer (-20 °C)

## Software and datasets

1. (Optional) Machine learning–based Organoids Analysis (MOrgAna; [20], v0.2.0, 2024), available for free as a GitHub repository at https://github.com/LabTrivedi/MOrgAna.git

## Procedure

**A. 2D induction**

1. Cell maintenance: Plating and passaging mESCs

*Note: For mESC maintenance, medium is changed every 2–3 days, and cells are passaged routinely once 70%–80% confluence is reached. If thawing cryopreserved cells, allow at least two passages before plating for inductions.*

a. Prewarm DMEM base (Recipe 1) and DMEM+++ media (Recipe 2) to 37 °C, and trypsin-EDTA (0.25%) to room temperature.

b. Coat a 6-well plate by adding 1 mL/well of 0.1% gelatin. Incubate at room temperature for 20 min with the lid off.*Note: Do not let the gelatin dry for longer, or cell attachment will be affected.*

c. Remove gelatin and add 2 mL of DMEM+++ medium per well.

d. Wash cells with 1 mL of PBS 1×. Aspirate.

e. Detach cells with 500 μL/well of trypsin-EDTA (0.25%). Incubate at 37 °C for 1–2 min.

f. Deactivate trypsin by adding 1 mL/well of DMEM base medium. Gently pipette with a 1 mL pipette to resuspend cells.

g. Transfer the cell suspension to a 15 mL conical tube and centrifuge at 137× *g* for 5 min at room temperature to pellet cells.

h. Aspirate the supernatant and resuspend the cell pellet in 1 mL of DMEM+++ medium.

i. Seed 100,000–400,000 cells per well. This typically corresponds to seeding with 5%–10% of the original volume.

j. Place the plate in a 5% CO_2_ incubator at 37 °C. Gently shake the plate back and forth and left and right to evenly distribute the cells. Do not disturb the plate for 24 h.

2. (**Day 0**) Seeding

a. Prewarm DMEM base medium to 37 °C. Prewarm NNE medium (Recipe 3) and trypsin-EDTA (0.25%) to room temperature.

*Critical: Always protect NNE medium from light.*

b. Coat a 12-well plate by adding 500 μL/well of 0.1% gelatin. Incubate at room temperature for 20 min with the lid off.

**Figure 1.**
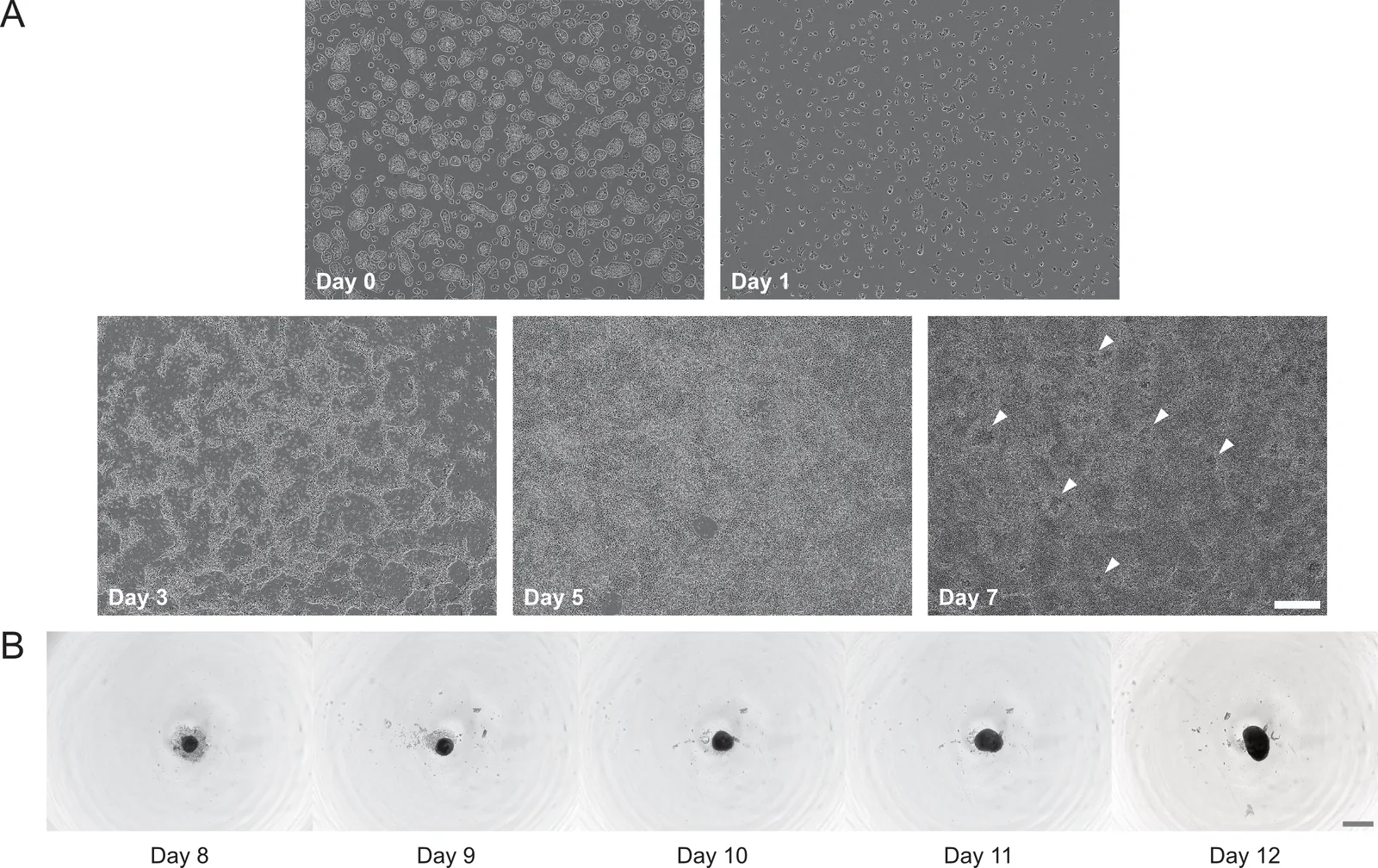
Representative brightfield images of the 2D induction and 3D budoid generation protocols. (A) Day 0 image shows mouse embryonic stem cells (mESCs) at the recommended 70%–80% confluence for seeding induction experiments. The remaining frames are taken from Video 1. White arrows indicate examples of dome-like structures formed by day 7. (B) Day 8 image shows an example of the expected cell shedding for the initial aggregate, which subsequently grows and elongates in one axis by day 12. Frames taken from Video 2. Scale bars, 500 μm.

*Critical: Do not let the gelatin dry for longer, or cell attachment will be affected.*

c. To set an induction, use mESCs that are 70%–80% confluent (Figure 1A). This protocol has been tested for mESCs up to passage 40, and knock-in lines up to passage 45. Wash cells with 1 mL/well of PBS 1×. Aspirate.

*Note: One well of a 6-well plate of mESCs at 70%–80% confluence yields >2 million cells—enough cells to set approximately 50 wells for heterogeneous induction.*

d. Detach cells with 500 μL/well of trypsin-EDTA (0.25%). Incubate at 37 °C for 1–2 min.

e. Deactivate trypsin by adding 1 mL/well of DMEM base. Gently pipette with a 1 mL pipette to resuspend cells.

f. Transfer the cell suspension to a 15 mL conical tube and centrifuge at 137× *g* for 5 min at room temperature to pellet cells.

g. Aspirate the supernatant and resuspend the cell pellet in 1 mL of NNE medium.

*Note: When passaging cells and setting a heterogeneous induction in parallel, the cells can be resuspended in PBS 1×.*

h. Stain 10 μL of cell suspension with 10 μL of trypan blue, transfer 10 μL of stained cells to a counting slide, and count the cells using an automated cell counter.

i. Prepare 1 mL/well of cells diluted to 40,000 cells/mL in NNE medium. Ensure cells are resuspended and use a serological pipette to seed 1 mL to each well of the 12-well plate. Pipette slowly against the side of the well.

j. Place the plate in a 5% CO_2_ incubator at 37 °C. Gently shake the plate back and forth and left and right to evenly distribute the cells. Do not disturb the plate for 24 h.

3. (**Day 1**) Media change

*Note: On this first induction day, the cells have just started differentiating, and some dead cells are to be expected (Figure 1A, Video 1).*

a. Prewarm NNE medium to room temperature.

*Critical: Always protect NNE medium from light.*

b. Aspirate media and wash cells with 1 mL/well of PBS 1×. Aspirate. See General note 3.

*Note: Handle gently, as cells are easily detached at this point.*

c. Add 1 mL/well of NNE.

*Note: Do this step at a time of day that can be kept consistent for all subsequent steps (see General note 4)*

**Video 1.**
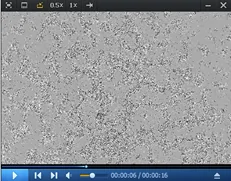
Representative brightfield time-lapse of the expected cell morphology changes throughout a successful mouse embryonic stem cell differentiation to limb bud–like cell types. Note that media changes took place at timepoints indicated in the video: 1 day 0 h, 3 days 3 h, and 5 days 1 h. Scale bar, 500 μm.

4. (**Day 3**) Induction medium I

*Note: At this point, protrusions from the differentiating cells should be visible (Figure 1A, Video 1). Expect a lot of dead cells. The morphology at day 3 tends to be predictive of the protocol’s success; if the colonies are sparse or if the plate is already confluent at this point, it is likely that the protocol will fail. Day 3 is also critical, as the cells are at their most fragile and can easily be washed off during media change.*

a. Prepare fresh induction medium I by supplementing NNE medium with 100 ng/mL BMP-4 and 1 μM SB 431542 (Recipe 4).

b. Aspirate media and wash cells with 1 mL/well of PBS 1×. Aspirate. See General note 3.

*Critical: Handle gently, as cells are very easily detached at this point.*

c. Add 1 mL/well of induction medium I.

5. **(Day 5)** Induction medium II

*Note: The cells should now have acquired epithelial-like morphology (Figure 1A, Video 1). Expect a lot of dead cells.*

a. Prepare fresh induction medium II by supplementing NNE medium with 100 ng/mL BMP-4, 100 ng/mL FGF-10, and 3 μM CHIR-99021 (Recipe 5).

b. Aspirate media and wash cells with 1 mL/well of PBS 1×. Aspirate. See General note 3.

c. Repeat step A5b for a total of two washes.

d. Add 1 mL/well of induction medium II.

6. **(Day 7)** End of induction protocol

*Note: The addition of BMP-4, CHIR-99021, and FGF-10 from the induction medium II introduces heterogeneity. By this final induction day, 2D cultures should have self-organized to form domes. There could be fibroblast-like cells, but the epithelial monolayer should be dominant in the culture (Figure 1A, Video 1). Each well of culture is expected to yield >800,000 cells.*

a. (Optional) Staining can be performed to confirm the cell types generated. For example, expect to see TP63 and *Fgf8* in AER cells, EPCAM in surface ectoderm cells, and *Prrx1* enhancer activity in limb bud mesoderm cells. Additionally, qPCR can be used where some genes, including *Wnt5a, Dlx5, Msx2, Fgf8, Wnt3*, and *En1*, can be expected to be significantly elevated compared to mESCs.

b. Proceed directly to budoid generation.

**B. 3D budoid generation**

1. **(Day 7)** Budoid generation

*Note: Budoids can be generated using mESC-based 2D induction cultures following section A, or by using in vivo–derived limb bud cells collected from mouse embryos. See General note 5. The seeding density of 9,000 cells in 100 μL per well is intended to yield a single budoid per well.*

**Figure 2.**
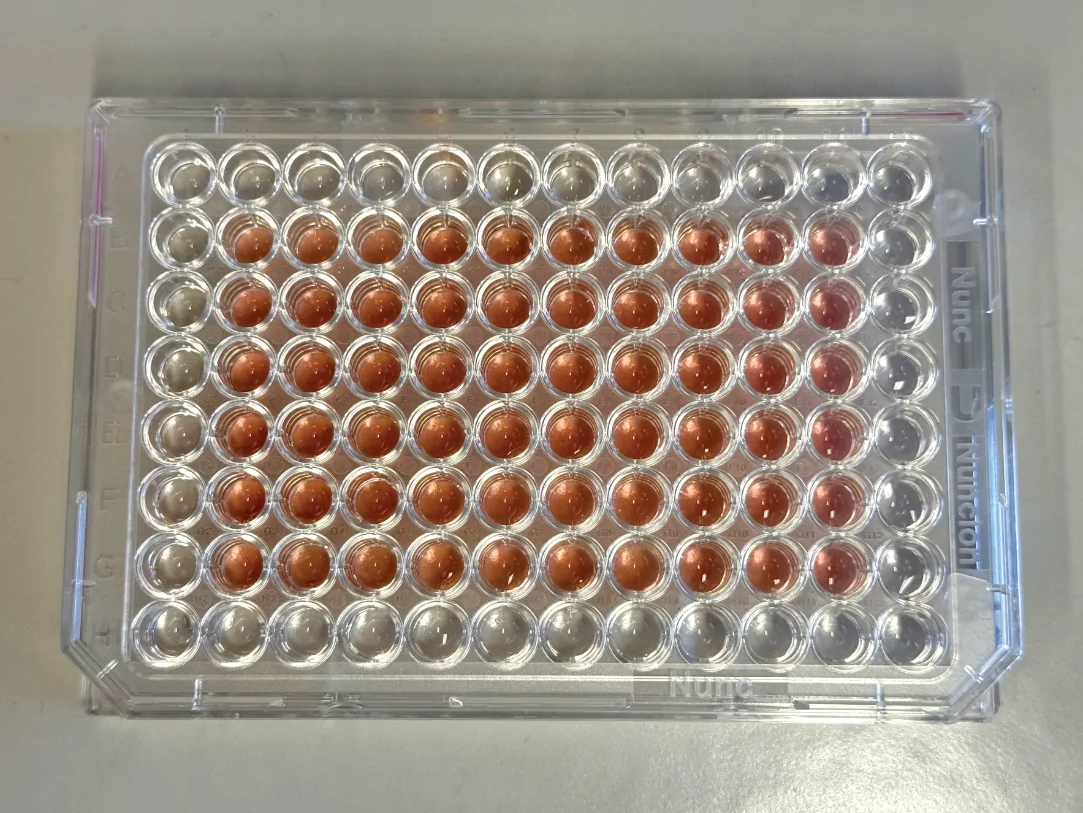
Recommended 96-well U-bottom plate layout for seeding budoids. Outer wells are filled with PBS, and budoids are seeded in only the inner 60 wells to minimize evaporation effects.

a. Prepare fresh budoid generation medium by supplementing NNE medium with 450 ng/mL FGF-8b and 300 ng/mL Wnt-3a (Recipe 6). Prewarm DMEM base to 37 °C and trypsin-EDTA to room temperature.

b. Prepare a 96-well U-bottom plate. As outer wells may experience more evaporation, budoids are seeded only in the inner 60 wells (Figure 2). Use a multichannel pipette and reagent reservoir to fill the outer wells with 200 μL/well of PBS 1×.

c. Aspirate media and wash cells with 1 mL/well of PBS 1×. Aspirate.

*Note: Each well of heterogeneous culture yields >800,000 cells, enough to set >80 budoids.*

d. Detach cells with 300 μL/well of trypsin-EDTA (0.25%). Incubate at 37 °C for 1–2 min.

e. Deactivate trypsin by adding 600 μL/well of DMEM base. Gently pipette with a 1 mL pipette set to 600 μL to resuspend cells.

f. Transfer the cell suspension to a 15 mL conical tube and centrifuge at 400× *g* for 5 min at room temperature to pellet cells.

g. Aspirate supernatant and resuspend cells in 1 mL of budoid generation medium.

h. Stain 10 μL of cell suspension with 10 μL of trypan blue, transfer 10 μL of stained cells to a counting slide, and count the cells using an automated cell counter.

i. Prepare 100 μL/well of cells diluted to 90,000 cells/mL in budoid generation medium. Transfer to 2 mL tubes and use a multichannel pipette to seed 100 μL to each inner well of the 96-well plate. Ensure that the volume is equal in all pipette tips and that the pipette is held straight and centered in each well, close to the bottom but without touching.*Note: A reagent reservoir can be used instead, but the final budoid elongation efficiency could vary between the two methods. It is recommended to either choose one method or test a side-by-side comparison.*

2. **(Day 8)** Budoids aggregated: Clear aggregates should now be visible. Expect some cell shedding (Figure 1B, Video 2).

**Video 2.**
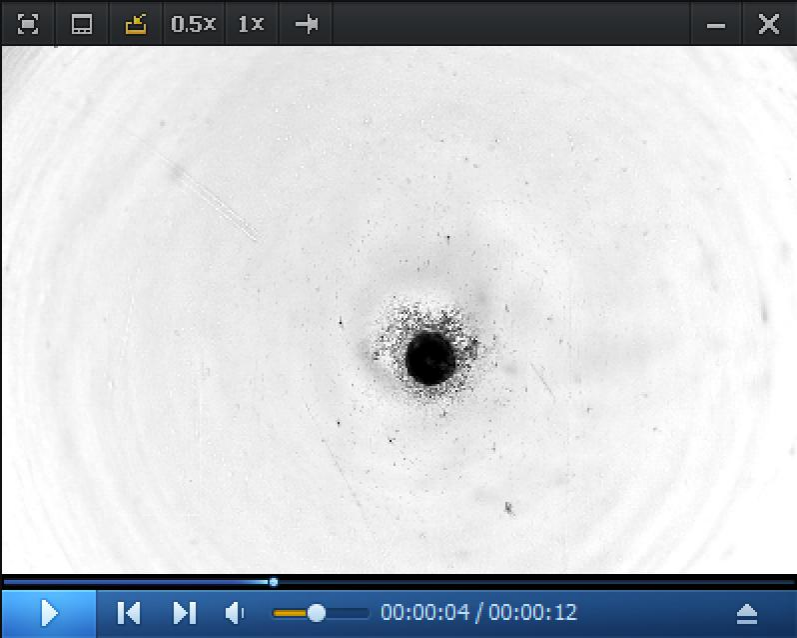
Representative brightfield time-lapse of budoid formation, growth, and elongation. Note that media changes took place at timepoints indicated in the video: 8 days 23 h, and 11 days 1 h. Scale bar, 500 μm.

3. **(Day 9)** Media change

*Note: At this point, budoids are growing but likely still spherical in shape (Figure 1B, Video 2).*

a. Prewarm NNE medium to room temperature.

*Critical: Always protect NNE medium from light.*

b. Use a multichannel pipette to carefully remove 50 μL of media from each well, touching the walls of the wells at an angle to avoid aspirating budoids. Add 50 μL/well of NNE gently against the sides of the wells. See General note 6.

4. **(Day 10)** Budoids may begin elongating: Some budoids may look slightly oblong already (Figure 1B, Video 2).

5. **(Day 11)** Media change

*Note: Budoid elongation should now be more apparent (Figure 1B, Video 2).*

a. Prewarm NNE medium to room temperature.

*Critical: Always protect NNE medium from light.*

b. Use a multichannel pipette to carefully remove 50 μL of media from each well, touching the walls of the wells at an angle to avoid aspirating budoids. Add 50 μL/well of NNE gently against the sides of the wells. See General note 6.

6. **(Day 12)** End

*Note: The majority of budoids should be elongated by the end of the protocol.*

a. Proceed to data analysis.

b. (Optional) Budoids can be collected for further downstream analysis, such as staining. As budoids are sticky, use low-attachment pipette tips with cut ends to gently transfer budoids into 1.5 mL protein low-binding tubes. If sticking is still an issue, pipette tips can additionally be coated in 1%–3% bovine albumin fraction V (BSA) in PBS 1×.

## Data analysis

**Figure 3.**
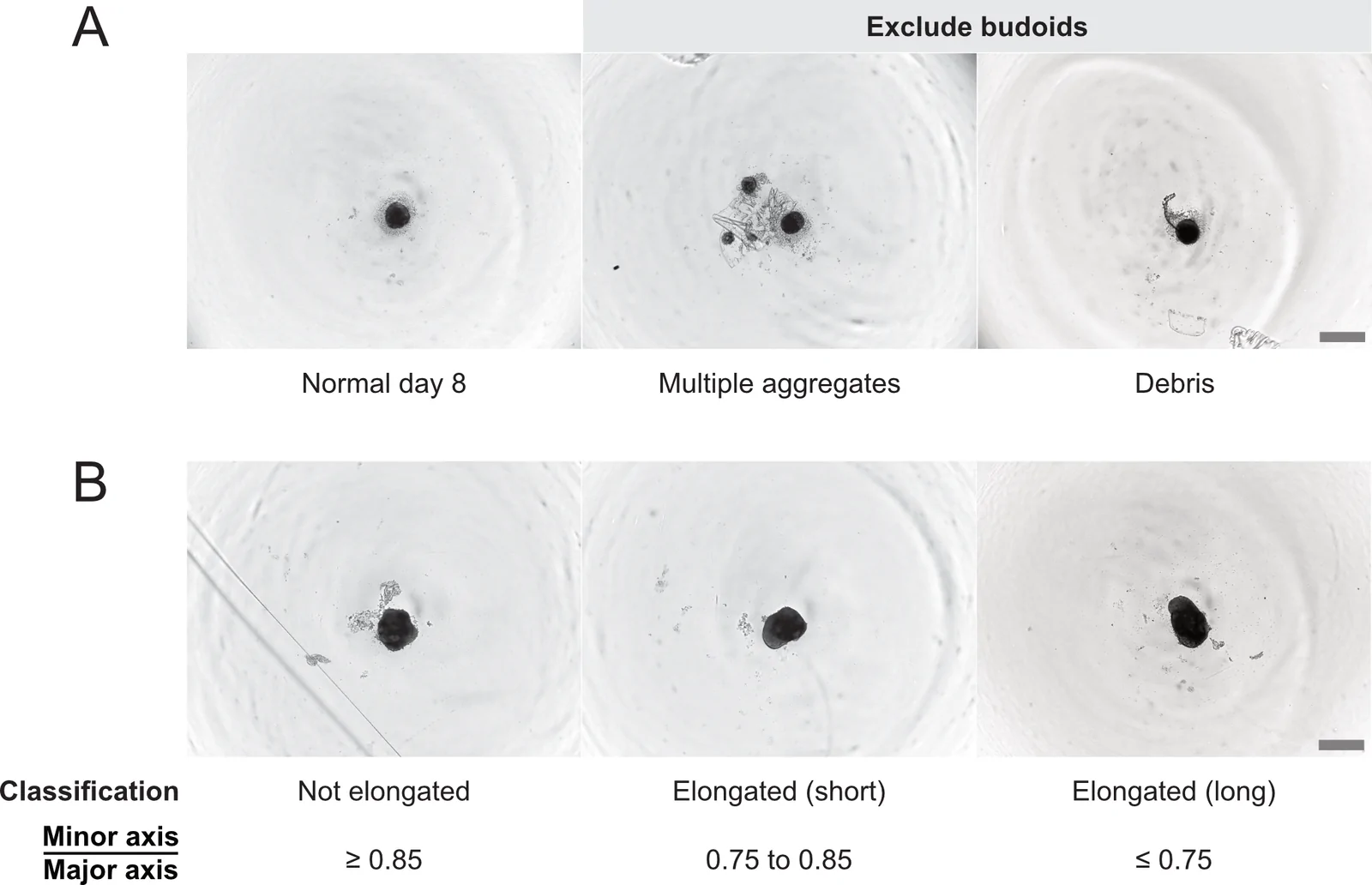
Representative brightfield images of budoid morphologies to be considered in data analysis. (A) Examples of exclusion criteria for day-8 budoids. (B) Elongation classification of day-12 budoids. Scale bars, 500 μm.

Any brightfield microscope can be used for analyzing budoid morphology. Days 8 and 12 are particularly critical for data inclusion/exclusion and experiment validation. On day 8, budoids are manually observed and excluded from further analysis if they show factors that could confound later assessments of elongation, such as the presence of multiple aggregates or debris (Figure 3A).

At the end of the experiment on day 12, budoid elongation is assessed based on the ratio of minor to major axis lengths. While no specific image analysis software is required, we used MOrgAna [20] to automate image segmentation and the quantification of budoid axes lengths based on the fit to an ellipse. As described in [21], we defined thresholds for classification: not elongated for (minor axis)/(major axis) ≥ 0.85, elongated (short) for 0.75 < (minor axis)/(major axis) < 0.85, and elongated (long) for (minor axis)/(major axis) ≤ 0.75 (Figure 3B). These thresholds were determined by manually classifying organoids by visual inspection, then checking the values that corresponded to these classifications. Across nine biological replicates, we found that 79.62% of budoids (168/211) fell into the elongated (short) and elongated (long) categories [21]. Therefore, an experiment is considered successful if it achieves similar elongation efficiency.

## Validation of protocol

This protocol has been used and validated in the following research article:

- Skoufa et al. [21] Specialized signaling centers direct cell fate and spatial organization in a mesodermal organoid model. *Science Advances* (Figures 1 and 2).

As described above in the Data analysis section, we check the elongation efficiency of the budoids to validate each experiment.

## General notes and troubleshooting

**General notes**

**1. Environment:** All cell culture should be performed under sterile conditions in a biosafety cabinet with sterile materials (purchased as such or autoclaved prior to use). Spray all items added to the biosafety cabinet with 70% v/v ethanol. All reagents and media should be prepared in the biosafety cabinet or sterilized with a 0.22 μm filter in the biosafety cabinet before use.

**2. Reagent preparation:** Reconstitute/dilute recombinant proteins and small molecules to the reagent concentrations indicated in the recipes. Follow the manufacturer’s recommendation for the specific batch, as it may vary. For most batches, we use ultrapure water to reconstitute LIF and FGF-8b at 1 mg/mL, and FGF-10 and Wnt-3a at 0.5 mg/mL, before diluting to 100 μg/mL with 0.1% BSA in PBS 1×. BMP4 is reconstituted in 5 mM HCl at 1 mg/mL, diluted to 100 μg/mL in 0.1% BSA in PBS 1×, and syringe-filtered through a 0.22 μm filter prior to aliquoting. For optimal stability, store as single-use aliquots at -20 °C for up to 3 months or -80 °C long term to avoid freeze-thaw cycles.

**3. Heterogeneous induction media changes:** To remove as many dead cells as possible, gently swirl the plate, tilt it slightly, and aspirate downward from the center of the well. Perform washes and media changes by pipetting gently against the side of the well, as cells can be damaged or washed off if the medium is dropped directly on top.

**4. Protocol timing:** For best results, perform all steps from days 1 to 12 within 48 ± 2 h of each other.

**5. Generation of budoids from in vivo mouse embryonic limbs**: It is also possible to generate budoids from in vivo–derived embryonic mouse limb cells. For this, dissect 10–15 hindlimbs and forelimbs of E12.5 mouse embryos into proximal and distal regions. Collect samples in protein low-binding tubes, wash with 1 mL of PBS 1×, and incubate in 300 μL of trypsin-EDTA (0.25%) at 37 °C for 5 min. Pipette up and down with a 200 μL pipette to dissociate the tissue and deactivate trypsin with 1 mL of DMEM base medium. Then, continue from step B1f.

**6. Budoid media changes:** To avoid losing budoids during media changes, hold the multichannel pipette at an angle touching the side of the well, and slowly aspirate the media. Check that the volume is equal in all pipette tips. Add new media by pipetting gently against the side of the well, as budoids can be damaged if media is dropped directly on top.

**Troubleshooting**

**Problem 1:** Cells washed off excessively during the heterogeneous induction; day 3 is particularly sensitive.

Possible causes: 1) Washes and media change not handled delicately enough. 2) Flawed gelatin coating.

Solutions: Swirl the plate gently and aspirate slowly for washes and media changes. Minimize the time the plate is left dry between aspiration and the addition of new media. When coating plates with gelatin, ensure that the entire well is covered and that the gelatin does not dry completely in the center of the wells during the incubation.

**Problem 2:** Inconsistent differentiation or budoid elongation between replicates.

Possible causes: 1) Variability between different lots of recombinant proteins and small molecules, particularly linked with BMP-4 and CHIR-99021, or changing any other materials. 2) High cell passage number. 3) Differing confluence of mESCs used for seeding on day 0.

Solutions: Use a single batch of materials for a set of related experiments. Concentrations of small molecules may need to be reoptimized for new lots. Keep a record of any product or lot number changes so potential problems can be traced back. Standardize an acceptable cell passage number range (e.g., we use our 129/SvEv mESC line until passage 40, and we have generated knock-in lines used until passage 45) and ensure that 70%–80% confluence of mESCs used for seeding is consistently defined.
